# Blogging with dementia: Writing about lived experience of dementia in the public domain

**DOI:** 10.1177/14713012221112384

**Published:** 2022-07-08

**Authors:** Jenni Brooks, Nada Savitch

**Affiliations:** Department of Psychology, Sociology and Politics, 7314 Sheffield Hallam University, Sheffield, UK; Independent Dementia Adviser, London, UK

**Keywords:** Dementia, blogging, writing, lived experience, living with dementia

## Abstract

Public narratives around dementia have historically been negative, and have not been shaped by people with dementia themselves, but stories of living with dementia are becoming more common in the public domain. This qualitative study explored the motivations and experiences of bloggers by conducting interviews with six bloggers with dementia in the UK. Thematic analysis suggested that the asynchronous nature of blogging offered a valuable, personalised space for people with dementia to tell their own stories in their own way. Blogging as a format posed some practical challenges, but bloggers developed strategies to overcome these. Motivations for blogging were on three levels: the personal (as a journal, and as a ‘room of one’s own’); community (as solidarity for other people with dementia, and as comfort for families and friends) and society (as an educational and campaigning tool). Whilst the study is small, and there are many voices of people with dementia missing from the blogging community, this research demonstrates the potential for blogging by people with dementia to change public narratives and perceptions of dementia.

## Introduction

Public narratives around dementia have often excluded the experiences of people with dementia themselves. Language around dementia has been largely medical, negative, or both, with words such as ‘suffering’, ‘burden’ on carers and the concept of dementia as a ‘living death’ still commonly used by the media (Peel, 2014). The condition has been seen at various points as a part of normal ageing, a solely biomedical issue or a relational problem, necessitating a focus on families providing care (Nedlund & Nordh, 2015; O’Connor & Nedlund, 2016). The voices of people with dementia have, in the past, largely been absent.

These negative narratives have been shifting in the last few decades. Tom Kitwood’s (1997) work around the idea of personhood has sought to shift away from an exclusively medical narrative to one which sees the *person* as well as the *illness*. The importance of hearing the perspective of people with dementia is now acknowledged in both research and practice (Bartlett & O’Connor, 2007), and activities such as life story work are frequently employed in social care and health settings (Gridley et al., 2016). In recent years there has been some acknowledgement of the decontextualised, apolitical nature of the concept of personhood, and a shift to focussing on citizenship, which locates the experiences of the person with dementia in their socio-political situation, and comprises (1) active participation by people with dementia in their own lives and wider society; (2) recognition of the potential for growth and positivity within the dementia experience; (3) understanding of the link between individual experiences and circumstances and broader socio-political and cultural dynamics and structures and (4) fostering solidarity between people with dementia (Bartlett & O’Connor, 2010).

Citizenship can be enacted through everyday talk and practice (Barnes et al., 2004), but for people with dementia, the ‘struggle for citizenship’ is ongoing (Bartlett, 2014). Citizenship is inherently linked with narrative – the personal, inter-personal and institutional/structural are linked ‘through the stories we tell and are told about us, whether by individuals or collectivities (such as the Law, businesses and government)’ (Baldwin, 2008, p. 224). The stories told about people with dementia are often medical, and medical narratives can overshadow all others, at least at first, and people can begin to interact with the diagnosis rather than the person (Sabat & Harre, 1992). Baldwin (2008) argues for the necessity of ‘narrative citizenship’, recognising that the opportunities for people with dementia to tell their own stories have been limited, but that these opportunities must be facilitated and nurtured if both personal and policy narratives are to change. People with dementia are increasingly involved in campaigning for social change (Bartlett, 2014), and there has been a proliferation of groups led by people with dementia, such as the Three Nations Dementia Working Group,^1^ Dementia Alliance International,^2^ and many of the groups which are part of the Dementia Engagement and Empowerment Project [DEEP] network.^3^ People with dementia have developed guidance for involving people with dementia in conferences and events (DEEP, 2013a), writing dementia friendly information (DEEP, 2013b), and the use of language for people writing about the condition (DEEP, 2015). Such examples encompass many of the elements of citizenship outlined by Bartlett and O’Connor (2010).

For many people with dementia, campaigning work begins by giving their own personal accounts of living with dementia. People with dementia have used a variety of platforms to tell their stories, including books (e.g. Mitchell, 2019, Swaffer, 2016 and see also Ryan et al, 2009, for an overview of the genre), discussion forums (Rodriquez, 2013) and Twitter (Talbot et al., 2020, 2021; Thomas, 2017). The Dementia Diaries project was created specifically for people with dementia to tell their stories easily – diarists use a 3D printed device (or their own telephone) to record their words, which are transmitted to the Dementia Diaries team, transcribed by volunteers and uploaded to the Dementia Diaries website (Woodall et al., 2016). Such innovations have demonstrated the value of online platforms to construct communities (Rodriquez, 2013), build self-esteem, support others and impact on policy and practice (Woodall et al., 2016).

The concept of telling stories about health and illness is not new. We must tell medical professionals, friends, employers and family what is wrong (or else make a choice to hide our maladies), and our stories may change depending on the audience. The stories that we tell are shaped by ‘all the rhetorical expectations that the storyteller has been internalising ever since he [sic] first heard some relative describe an illness’ (Frank, 2013: p3).

Health blogging – writing blogs about health conditions – is also relatively common. Academic research has explored the blogging activities of women with breast cancer (Martino et al., 2019; McNamara, 2007), burns survivors (Garbett et al., 2017), women with endometriosis (Fernley, 2021), people with depression (Kotliar, 2016), as well as health blogging more generally (see, for example, Rains & Keating, 2011). Health blogging has been examined as a means of providing social support for people with health conditions (Rains & Keating, 2011), and has been associated with modest improvements in wellbeing (Rains & Keating, 2015), and there is a suggestion that reading blogs written by someone with personal experience of a health condition (in this case HIV) are more persuasive in changing action than blogs written by professionals (Neubaum & Krämer, 2015).

In people who care for people with dementia, blogging has been shown to reduce stress (Hori et al., 2010) and foster community (Anderson et al., 2016). Carers’ blogs have explored the concept of dignity for people with dementia (Anderson et al, 2021); needs, concerns and advice of caregivers (McLennon et al., 2021); and suicide and homicidal ideation in family carers of people with dementia (Anderson et al., 2019). However, this work has all been from the perspective of the carer – the perspective of people with dementia themselves is absent.

Blogging as a medium for people with dementia themselves to tell their own stories has been largely neglected in academic research. One exception to this is a study by Kannaley et al. (2019), which analysed five posts from blogs written by both people with dementia and carers and identified a series of themes, including seeing the positives, feeling out of control, advocacy and empowerment and coping mechanisms. However, their analysis did not separate blogs written by people with dementia from those written by carers. In this paper we use a citizenship perspective to explore the motivations of people with dementia to write their own blogs, and the practical challenges of blogging for people with dementia.

## Methods

The findings in this paper are taken from a small scale qualitative study exploring blogging by people with dementia, funded by Sheffield Hallam University and conducted in 2017 and 2018. The study was led by the researcher (Jenni Brooks), with Nada Savitch (an independent dementia adviser) advising on and supporting the involvement of people with dementia.

### The study had two stages

 (1) Interviews with people with dementia in the UK who write their own blogs.
(2) Analysis of blogs written by people with dementia (in English) across the world.

This paper reports the first of these two stages. The aim for this stage was to explore motivations and experiences of UK bloggers with dementia, and to examine their perceptions of the benefits, challenges and practicalities of blogging.

### Recruitment and participants

Recruitment focused on bloggers with dementia, based in the UK, who wrote sole-authored blogs (not hosted by organisations or with multiple authors). Existing lists of bloggers with dementia provided a starting point,^4^ followed by an internet search for ‘blogger with dementia’, and ‘dementia blog’, and by snowball sampling.

Blogs were only selected for inclusion if they

  • were written by people with dementia (based on their self-identification on the blog) living in the UK;
  • were published by individuals rather than organisations;
  • were publicly available and
  • had posts within the previous year.

Saturation was reached after identifying eight blogs, with each new search only returning the same results, and each blogger only pointing towards the same pool of others.

We contacted each blogger using the contact form or email address from their blog, or where these were not available, their public Twitter profile, inviting them to take part. Information about the project was written in accordance with the DEEP guidelines for good communication with people with dementia (DEEP, 2013b). Seven out of eight bloggers responded, one declined to take part, and six agreed. All participants were able to read and understand the information leaflet and give written informed consent to take part.

Table 1 shows the characteristics of the participants.

**Table 1.** Characteristics of participants.

Name	Length of time blogging before interview	Age at diagnosis
Carol	6 weeks	58
George	2 years 11 months	63
Ken	11 years	56
Valerie	2 years 11 months	Late 50s
Wayne	1 year 2 months	59/60
Wendy	2 years 9 months	58

All six participants were white, British and had been diagnosed with dementia between the ages of 55 and 63. All had some degree of computer literacy, but none had previous experience of blogging, although one (Valerie) was a professional author, and another (George) had an English degree and was used to writing reports in his previous job.

### Data collection

Participants were supported to take part a way that suited them. Three participants chose to be interviewed at home, and the other three chose public locations – two cafes, and one hotel lobby. Two participants had spouses present, another spouse was in the house but not the same room, and three participants were interviewed alone.

All participants were sent a list of interview topics in advance – one requested a detailed list of questions to prepare for, which was sent. The interview schedule included questions about reasons for starting blogging, reading other people’s blogs, frequency and topics of blog posts, topics avoided in blog posts, perceptions of blog readers, positive and challenging aspects of blogging, support from other people, changes over time (including perceived future changes), and advice for other potential bloggers with dementia.

The day before each interview the researcher called to confirm. On arrival, we talked through the project information again, and each interviewee gave written informed consent. Interviews lasted 1–2 hours and were audio recorded and later transcribed. Each participant was given a £20 gift voucher to say thank you.

### Analysis

Thematic analysis was carried out using the Framework approach (Spencer et al., 2013) to manage interview data. The Framework approach is a matrix-based method which allows qualitative data to be organised and synthesised consistently, supporting identification of themes both within and across cases. It has advantages in allowing later stages of analysis to be directly connected to original interview quotes to test emerging findings in the context of the whole dataset. The researcher (Jenni Brooks) transcribed all interviews, re-read for familiarity, and developed a coding framework including a priori themes from the topic guide and emerging themes, which was discussed with Nada Savitch. Jenni Brooks then extracted interview data into the framework and assigned index categories, then created a series of central charts summarising and synthesising the data, using participants’ own words wherever possible. Jenni Brooks then conducted an iterative process of mapping and interpretation to make sense of the data, and discussed and clarified this with Nada Savitch.

A short summary of findings, again prepared using the DEEP (2013b) guidelines for written communication with people with dementia, was sent to each participant following analysis. Findings were also presented at the UK Dementia Congress in 2018 to an audience of practitioners, academics and people with dementia, including two of the study participants.

### Ethical considerations

Ethical approval for this project was granted by Sheffield Hallam University Faculty of Development and Society Ethics Committee on 26^th^ May 2017 (ref: 394-BRO).

Each participant was asked whether they wanted to choose a pseudonym, but all asked to appear in the research under their own name. This is consistent with Bartlett’s (2014) research with people with dementia who campaign for social change.

### Findings

The aim of the study was to explore the motivations and challenges experienced by people with dementia when writing their own blogs. This section begins with a brief outline of what makes blogging as a format valuable for people with dementia, followed by a discussion of how participants overcame some of the practical challenges of blogging. The main body of the findings section focuses on motivations for blogging, and these are grouped under three broad themes: the personal level (blog as journal, blog as a ‘room of one’s own’); community level (blog as solidarity, blog as comfort) and society level (blog as education, blog as campaign tool).

### Blogging as a format

The format of blogging allowed bloggers to tell their stories in ways they may not have otherwise been able to do, but there were still practical challenges which they had developed strategies to overcome.

The asynchronous nature of blogging was perceived as a particular benefit for people with dementia, allowing bloggers to draft posts over the course of days, sometimes weeks, before publishing them online, and all took advantage of this, often writing drafts in a word processing programme. This allowed them to collect their thoughts before writing.

‘I’m not so good at speaking off the cuff…. [Blogging] gives me time to reflect, to think out exactly how I want to say something’ (George).

In this way, the bloggers could join in public conversations about, for example dementia policy or human rights, without having to respond quickly, which they often found difficult.

All of the bloggers had used other forms of written online communication (for example social media and forums) since their diagnosis to communicate with other people with dementia, but blogs were seen as a personal space, where they retained control, and could decide what to write about and how to present their thoughts, with no word limit. All had gone some way to personalise their blog space by choosing templates, or using photographs.

### Overcoming practical challenges

Blogging as a format allowed bloggers to tell their stories in ways they may not have otherwise been able to do, but it did pose challenges, which the bloggers had developed their own strategies to overcome. Several wrote drafts of posts over a number of days before copying them onto the blog. Some found the blogging software interface difficult to navigate, and one had asked someone else to set up a shortcut on the desktop of their computer to take them directly to the ‘new post’ page. Carol created her own personal ‘helpdesk’ of instructions for regular tasks and useful phrases that she could easily find when she needed them.

Wayne described how a friend acted as informal ‘editor’.

‘Sometimes I get angry! [laughing] And… I see everything very black and white, that’s the dementia… and Dave has to try and tone it down, so we’re keeping the frustration there, but taking the explosive anger out!’ (Wayne).

Wayne was unique in this sense though – the other bloggers occasionally asked other people to read a particular post but did not have this editorial relationship. People did have other strategies though, such as keeping a bank of words and phrases on the computer, and keeping an ongoing list of posts for when they did not have any idea what to write about.

All of the bloggers were candid in their acknowledgement that their ability to keep writing their blog would likely change in the future and some were already altering their blogging habits and strategies. Some, finding typing difficult, had tried voice recognition software, with mixed success. Others found themselves getting frustrated with blogging software, and writing less as time went on. Valerie felt she was coming to the end of her blog, found using Wordpress ‘murder’, and felt ‘I really cannot now do something so long and purposeful’. Similarly, Wayne found his posts taking longer, and joked: ‘I’m spending so much time living life I haven’t got time to write about it any more’. Most, though, were determined to continue blogging whilst they could.

The rest of this findings section deals with the reasons the bloggers gave for blogging.

### Personal motivations – blogging for myself

There were two sub-themes at the personal level: the blog as a journal – a place for people with dementia to capture their thoughts and memories; and the blog as a virtual ‘room of one’s own’, a space for bloggers to exert autonomy and control, and in which to find their own voice.

#### Blog as journal

Several of the bloggers already had some involvement with dementia-related groups and started their blogs partly to keep a record of their activities. Wendy, for example found that ‘exciting things were happening, and they were getting lost’, and for her, looking back at her own written and photographic accounts of events and activities was ‘quite therapeutic… whether you’ve forgotten it or not doesn’t matter’. Carol’s experience was similar – one of the reasons she was writing was

‘to build a memory book that I can look back on further down the line, reading about all the things I’ve done… I’m hoping it’s going to be like a morale booster, you know, oh I did all those things’ (Carol).

In this sense the blogs were being used almost as contemporary life story accounts, documenting each activity as it was happening or shortly after. Wendy in particular described how she often wrote blog posts about an event during the event itself, and in fact wrote the outline of a post about being interviewed for this study during the interview.

For others, blogs functioned more as personal ‘stream of consciousness’ style journals rather than an account of activities. For example for Valerie, whose long career as a writer predated her blog by several decades, her blog was

‘like a diary really of my progress, just thoughts and feelings and the odd poem. And there was no structure to it whatsoever, none, it was just an outlet, there was nothing generous about it, it was a me, me, me thing, it was just for me’ (Valerie).

Despite this, Valerie did intentionally publish her blog online, unlike Ken, who was advised to keep a diary by a clinical psychologist as a way of dealing with his graphic nightmares, and did not realise his electronic journal was public for 18 months. After the initial shock, Ken read the comments on his blog and decided to keep it public – this is discussed further in the ‘blogging as reassurance’ section below.

Wayne’s blog also began as a series of private notes, made about events he had attended, in an attempt to keep the appearance of having a good memory. Other people recognised the value of these notes, and in time, Wayne began to turn them into a blog. These shifts from private (or at least intended to be private) journals to public resources demonstrates the value other people place on accounts written by people with dementia.

There was also a sense that these written accounts might be useful in supporting person-centred care in the future.

‘Eventually you reach a point on your journey when you’re not capable of looking after yourself and somebody else is going to have to do it. Well for somebody to be able to look back on a blog that... is not quite day by day, but event by event expression of who you really were, that’s still inside... that’s brilliant’ (Wayne).

In this sense, blogging shares some characteristics with life story work.

#### Blog as a room of one’s own

The blogs were autonomous virtual spaces, where their authors made decisions about tone, style and content. Aside from Ken, setting up a blog had been a deliberate act for all the bloggers, and in some cases they had spent considerable time reading other people’s blogs, and defining the tone of their own.

Valerie’s experience differed in some ways to the other bloggers. She had been a writer for many years, and for her, the blog was just one outlet among many through which to continue her writing: ‘Little words still flow through me, little sentences…’. She felt sharing these snippets of thoughts and poetry would be more beneficial for her than sharing too much of ‘me and my doom and gloom’, because

‘I think that would have a negative impact on me, because I try and work out all the things I can do rather than the things that I can’t’ (Valerie).

The other bloggers did also share positive experiences and other elements of life, but were more specifically focused on sharing about their experience of dementia.

All of the bloggers were very aware of issues around privacy, and the impact of their writing on other people, and had given considerable thought to what they were willing to share. For example some, like George, did not write about their own medical appointments, whereas others were quite candid about their health, but did not mention family members or personal relationships.

The asynchronous nature of blogging gave people with dementia the ability to join in ongoing conversations in a way that they may otherwise have been unable to, as dementia can make speaking in public, particularly in group settings, more challenging. In this way, blogging made people feel 'normal’ again.

‘I can type quicker than I can think and speak the words. Because that part of my brain hasn’t broken, typing just makes me feel normal again. I can do it far better than talking to people’ (Wendy).

Each blogger set out practical strategies for facilitating their writing, such as drafting and editing over several days, or having a friend read a post before publishing.

### Community motivations – blogging for people with dementia and their loved ones

Several bloggers had started their blogs with an explicit aim of providing support to others, and all listed this as one of their motivations to continue writing. This support took two forms: solidarity with other people with dementia, and reassurance for family and friends.

#### Blog as solidarity

Bloggers talked about using their blogs to demonstrate to other people with dementia that life could continue after a diagnosis – ‘to show that you can continue to lead a normal, active, well, not a normal life, but an active life’ (Carol).

Some people mentioned how scared they themselves had been after their own diagnosis. Meeting other people with dementia and reading their words had helped them see they could continue to live a good life and they wanted to pass this encouragement to other people with dementia, to let them know

‘that life doesn’t end with a diagnosis, that it opens up a different world, and it’s just all about adapting and doing things differently, but there’s still lots out there to be done’ (Wendy).

The bloggers treated their work in this area seriously, and considered themselves part of a peer network providing support to other people with dementia. The online element of blogging was thought to be valuable in this respect, as after diagnosis, ‘people very quickly don’t go out, they lose their confidence, and they sit and home and watch tv’ (George). Having online access to the experiences of other people with dementia was therefore a way of gaining a sense of community, without being compelled into a face to face social situation.

‘We live in our little individual bubbles, and it’d probably be the same for any other disease as well, but it’s really important to know that we’re not alone’ (George).

It was this that had encouraged Carol to begin writing her own blog.

#### Blog as reassurance for families

The bloggers also found that they were providing comfort and reassurance to people whose relatives were living with dementia. For example Ken first realised his blog was public when he clicked on a ‘comments’ box 1 day and found

‘about 200 comments from people, all over the world, saying thank you for this, because you’ve explained so much the doctors won’t tell us’ (Ken).

Ken was initially ‘terrified’ at this discovery, but decided to continue writing, because ‘it’s humbling to think you’re helping somebody’ – a sentiment shared by other bloggers too. This help could take a form that others without dementia were unable to give:

‘Often people who’ve had relatives with dementia who died, they’re saying oh I now realise why they did something, because of something I said’ (Wendy).

### Societal motivations – blogging for healthcare professionals and wider awareness

The bloggers’ concept of helping people extended beyond other people with dementia and their families to encompass two other areas: education for healthcare professionals and others working with people with dementia; and campaigning for change in wider society.

#### Blog as education

None of the bloggers started with an explicit aim of educating healthcare professionals, although some found themselves doing just that. Several had been invited to give talks to medical students, and Ken described how his blog was being used as a ‘teaching tool’ at several universities, including his most local one, where nursing students were encouraged to search for specific topics on his blog, such as 'waking people up from graphic nightmares’.

Several bloggers acknowledged their ability to communicate about dementia was not something necessarily shared by others. For example

‘Many people may not be quite as far on the journey, but have had their communication centres attacked, and are less able to communicate… what I was therefore putting across in words were things that quite often they don’t get chance for the person living with dementia to tell them…. So it very quickly proved to be a useful tool’ (Wayne).

Bloggers therefore saw writing about their experiences of dementia as a service which could help healthcare professionals and others better understand dementia.

#### Blog as campaign tool

Some of the bloggers had deliberately started their blog as a campaigning tool, mostly to complement other campaigning activities they were taking part in, for example as part of the Dementia Action Alliance.

‘I’m writing for an audience, I’m not writing just because I want to write, I could do that on a piece of paper… I’m writing as an activist, I want people to read it and re-examine what they think and what they do’ (George).

Even those who had primarily started blogging as a journal for themselves later came to recognise the importance of the platform for raising awareness about the realities of living with dementia, including medical appointments and accessing services and support. In some cases, bloggers had been invited to speak at conferences or to the staff of organisations about their experiences.

## Discussion

This paper argues that blogging by people with dementia expands the range of public stories that are told about the condition, and contributes to the enactment of ‘narrative citizenship’ for people with dementia through addressing the personal, inter-personal and institutional/structural elements and the relationships between them (Baldwin, 2008). Blogs as a format do offer something different to other online platforms that is particularly valuable for people with dementia. Blogs are asynchronous, so thoughts can be formed and written over time, meaning people with dementia can avoid problems of ‘outpacing’ (Kitwood, 1997). The bloggers in this study valued being able to write a blog post over several days; add photographs either to enhance, or when they found words more difficult; change themes or colour schemes to personalise their blogs and write as often and as much as they chose. There is no obligation to take part in a conversation as there may be with a discussion forum, and their writing appears together, rather than interspersed between the writing of others, giving them an opportunity to create a coherent narrative. Being in control of the environment limits the potential for encountering hostile people, as is a possibility on social media (Talbot et al., 2021).

People with dementia used their blogs to facilitate communication with, and support for, other people with dementia, echoing the use of other forms of online communication, such as Twitter (Talbot et al., 2020; Thomas, 2017), internet discussion forums (Rodriquez, 2013) and the purpose-built Dementia Diaries platform (Woodall et al., 2016).

The long form nature of blogging allows for an enhanced level of insight into the experiences of living with dementia, and bloggers identified that this was of benefit to themselves, to other people with dementia, family and friends, as well as health professionals and the general public. Bloggers chose what to write about, what news stories and policy decisions to respond to, and made specific and nuanced decisions about their own privacy. There has been an increased recognition of the need for people with dementia to be included in research (Dupuis et al., 2012; McKeown et al., 2010; Murphy et al., 2015), and in the education of healthcare professionals (Cashin et al., 2019), but blogging gives them a way to set the agenda, to talk about what is important to them – not just respond to the specific concerns of policy makers and professionals. Other research has demonstrated that reading blogs written by someone with personal experience of a health condition is more persuasive in changing action than blogs written by professionals (Neubaum & Krämer, 2015).

Many of the bloggers in this study also considered themselves to be campaigners, and blogging, for them, shared some similarities with campaign work by people with dementia (Bartlett, 2014), in some senses being considered campaigning in itself. Unlike other forms of campaigning, for example speaking in public, blogging allowed the bloggers time and space to articulate their points of view, the challenges faced in sharing personal experiences in public, and the difficulties involved in overcoming these challenges. These blogs, as longer form narratives accessible to be read by other people, go some way to bridging this gap between experience and understanding, making this emotional, and sometimes physical, ‘backstage’ labour visible. This is the ‘backstage’ work that participants in Bartlett’s (2014) study found often went unrecognised by audiences.

This presentation of the ‘backstage’ work involved in telling personal stories of dementia in public is important. In recent years, people with dementia have been ‘increasingly finding both a voice and a narrative space’ (Baldwin, 2008, p. 225). However, this can come at a cost, as demonstrated by many of Bartlett’s (2014) participants having their diagnoses questioned as they did not ‘behave in a way that one might “expect” a person with dementia to behave, (p. 1300). The bloggers in this study are giving extensive, ongoing accounts of the realities of living with dementia, sharing both positive and negative experiences. By telling their stories slowly, in their own time, and their own voice, they are increasing the variety of public narratives of what it means to live with dementia.

Blogging as an activity is, of course, not available to everyone. Income, age, household composition, mobility and memory are all contributors to digital exclusion (Age UK, 2018). Internet use is increasing in all age groups, but in 2018, over half of all adult internet non-users were over the age of 75 (Office for National Statistics, 2019). There are moves towards more technological interventions for people with dementia, for example the use of iPads in care homes (Evans et al., 2017), but specific interventions mediated by staff members are very different to independent blogging. There is evidence that more older people began using the internet during the Covid 19 pandemic (Ageing Better, 2020), but no evidence about internet use by people with dementia.

This study is small by nature. At the time of participant recruitment, only eight bloggers with dementia were identified in the UK, and six of those agreed to interview. The bloggers included in this study all lived in England, spoke English, were white, did not live in residential care, and had some support from family or friends. They were all diagnosed with some form of young onset dementia between the ages of 56 and 63. None of them had previous blogging experience, but they had all had some degree of computer literacy.

Voices are still missing from the blogging community in England, and therefore from this research. The experience of dementia is very different for people with different ethnic backgrounds (Moriarty et al., 2011), who are not represented here, and according to sexuality (McGovern, 2014) and socio-economic status, which this study did not collect data about. Older and more frail people with dementia are missing from the blogging space, and therefore from this study, too. Similar omissions were found in research about the use of Twitter by people with dementia (Talbot et al., 2021).

It is unsurprising that relatively young, computer literate people might take to blogging about their experiences of dementia in greater numbers than people who are older, have little experience of computer use throughout their life, or are more frail and therefore perhaps unable to use such technology without support. People with dementia who are involved in campaigning work more widely tend to be relatively young and healthy (Bartlett, 2014). The voices of people with dementia have been absent in the public narrative for so long that any are a welcome addition and contribute to the public understanding of living with the condition. However, the relative demographic homogeneity of bloggers with dementia, does mean that the variety of stories available in this space is limited. There is a risk that those with different experiences of living with dementia remain unheard.

## Recommendations

Blogging is a personal activity for a public audience. This study has identified that bloggers are motivated at the personal, community, and societal level to share their stories, but not everyone with dementia will want to share their thoughts online. When they do, there may be some value to technical support from other people. Some studies have started to explore the specific needs of internet users with dementia, particularly in relation to website design (Freeman et al., 2005; Schnelli et al., 2021), and if implemented by blogging software providers this may go some way to reducing the technological challenges experienced by some of the participants in this study. It is also possible that existing bloggers without dementia may develop the condition in the future and continue their blogging activities, leading perhaps to a wider range of voices and subjects written about by people with dementia.

Writing in general may be a useful way for people with dementia to ‘grow positively with dementia’ (Ryan et al., 2009: p. 156), and blogging can be beneficial for the writer themselves, for readers, and for education and raising awareness in the wider community. Writing a blog does not require the commitment of writing a book, and provides some of the social and community benefits to the writer themselves of other forms of online communication, whilst shielding them from some of the more negative aspects associated with some forms of social media (Talbot et al., 2021). It is tempting to recommend that blogging be attempted more widely by people with dementia, but one of the key motivations for blogging is being able to share personal stories in a personal way, so editorial control should remain with the blogger.

Blogs blur the boundary between public and private space (Snee, 2013) and there are potential ethical issues in using them in a way not intended by the author. It may be useful for professionals to read blogs written by people with dementia to gain an insight into the everyday reality of living with the condition, and to hear experiences that may not be heard in healthcare settings – but they should be mindful that there still are voices and perspectives missing, and not assume that the stories are representative. For the bloggers in this study, raising awareness among professionals was an important outcome of blogging. However, anyone using a personal blog in a professional context should be mindful of the author’s intentions, perhaps following Whiteman’s (2010) suggestion to check the author shows an awareness of unknown readers in their writing.

## Conclusion

This study has explored the motivations and challenges faced by bloggers with dementia. We have found that blogging as a format offers benefits particularly welcome to people with dementia, and that bloggers with dementia have found creative ways to overcome technological challenges of blogging. Motivations for blogging exist at an individual level (blogging as a journal for personal thoughts, and as a personal online space); at a community level (as solidarity for others living with the condition, and comfort for family and friends), and the society level (as education and a campaign tool). By working in these spheres, blogging can enable the enactment of some elements of active social citizenship for people with dementia.

Bloggers with dementia are challenging assumptions about what people with dementia are capable of, and are creating new stories of what it means to live with dementia which can enter the public consciousness.

We should be mindful of the implications for other people with dementia. Telling one’s story publicly should never become compulsory, but there is potential for others with dementia, for family and friends, and for healthcare professionals, to read and learn from the lived experience of bloggers with dementia to gain a better understanding of what it is like to live with the condition, and to ultimately shape better care.

## Notes

1 Three Nations Dementia Working Group: https://www.3ndementiawg.org/

2 Dementia Alliance International: https://www.dementiaallianceinternational.org/

3 DEEP network: https://www.dementiavoices.org.uk/

4 Dementia Action Alliance – https://daanow.org/resource-center/blogs/; Young Dementia UK – https://www.youngdementiauk.org/resources/blogs; Healthline – https://www.healthline.com/health/alzheimers-disease/best-blogs-of-the-year
